# Embryonic Stem Cell (ES)-Specific Enhancers Specify the Expression Potential of ES Genes in Cancer

**DOI:** 10.1371/journal.pgen.1005840

**Published:** 2016-02-17

**Authors:** Dvir Aran, Monther Abu-Remaileh, Revital Levy, Nurit Meron, Gidon Toperoff, Yifat Edrei, Yehudit Bergman, Asaf Hellman

**Affiliations:** 1 Developmental Biology and Cancer Research, The Institute for Medical Research Israel-Canada (IMRIC), The Hebrew University-Hadassah Medical School, Jerusalem, Israel; 2 Institute for Computational Health Sciences, University of California, San Francisco, California, United States of America; University of Toronto, CANADA

## Abstract

Cancers often display gene expression profiles resembling those of undifferentiated cells. The mechanisms controlling these expression programs have yet to be identified. Exploring transcriptional enhancers throughout hematopoietic cell development and derived cancers, we uncovered a novel class of regulatory epigenetic mutations. These epimutations are particularly enriched in a group of enhancers, designated ES-specific enhancers (ESSEs) of the hematopoietic cell lineage. We found that hematopoietic ESSEs are prone to DNA methylation changes, indicative of their chromatin activity states. Strikingly, ESSE methylation is associated with gene transcriptional activity in cancer. Methylated ESSEs are hypermethylated in cancer relative to normal somatic cells and co-localized with silenced genes, whereas unmethylated ESSEs tend to be hypomethylated in cancer and associated with reactivated genes. Constitutive or hematopoietic stem cell-specific enhancers do not show these trends, suggesting selective reactivation of ESSEs in cancer. Further analyses of a hypomethylated ESSE downstream to the VEGFA gene revealed a novel regulatory circuit affecting VEGFA transcript levels across cancers and patients. We suggest that the discovered enhancer sites provide a framework for reactivation of ES genes in cancer.

## Author Summary

We demonstrate that a particular subtype of transcriptional enhancers, which we termed ES-specific enhancers (ESSEs), are especially prone to DNA methylation perturbations in cancer. Moreover, the methylation status of ESSEs, but not that of the enhancers of tissue stem cells, is associated with gene activity in cancer cell lines and in fresh tumor samples. Extending the previous reports on the hypermethylation of particular gene promoters in cancer, our analyses revealed both hyper- and hypo-methylation of regulatory elements in cancer, in association with silencing and enhancing, respectively, of important cancer genes. Thus, the discovered sites are appealing candidates for epigenetic control of cancer expression profiles.

## Introduction

Disruption of normal cellular identity is a hallmark of cancer cells [1]. Various cancer types display gene expression profiles that show similarities to the expression program of embryonic stem cells (ES), but are absent from the differentiated tissue from which the cancer evolves. These ES and cancer-specific expression signatures comprise genes of the ES core module, including NANOG, OCT4, SOX2 and KLF4, the polycomb repressive complex 2 (PRC2) module including EZH2, EED, and SUZ12, the MYC module and other genes [2–6] (reviewed in [7]). Many of these genes participate in inter- and intra-cellular signaling pathways. ES-like gene expression profiles may result from re-wiring of stem cell regulatory circuits in cancer. In line with this theory, DNase I hypersensitive sites profiling revealed extensive reprograming of ES-specific regulatory elements in cancer [8]. Moreover, ES-like chromatin patterns mediated by the PRC2, which predispose genes to DNA hypermethylation and permanent silencing in cancer, have been reported in loci proximal to transcription start sites (TSSs) [9,10]. However, the regulatory mechanisms underlying the establishment of ES-related expression profiles in cancer are not well understood.

Transcriptional enhancers control the expression of genes independently of their orientation, position or distance relative to TSS [11,12]. Their genomic locations, covering more than 10% of the human genome, are readily detected on the basis of characteristic chromatin marks, primarily the monomethylation of histone 3 lysine-4 (H3K4me1) [13,14]. Many enhancers are specific to certain tissues or cell types, including a particularly large group of enhancers that are exclusive to embryonic and induced pluripotent stem cells [15–17]. In contrast to gene promoters, which tend to maintain their core chromatin structure across expressing and non-expressing cells, with some minor activity-related modifications, enhancer chromatin is not constant across cell types. Rather, enhancer sites may display active, inactive, or entirely naïve non-regulatory chromatin states across cells. The generation of the enhancer chromatin landscape is a complex multilayered process that involves the recruitment of chromatin modulators and pioneer transcription factors to certain genomic sites upon specification of particular cell lineages. Once established, enhancers can be found in active or inactive states, characterized by H3K27ac and H3K27me3, respectively, and by other chromatin features. However, in other cell lineages the very same sites do not display regulatory chromatin structures, and are unable to support transcription [18–21]. Interestingly, epigenetic reprograming of enhancer elements was previously shown to be prevalent in multiple cancer types, and evidence showing their role in cancer development has been reported [22–24]. However, the rules that govern the activation or silencing of these regulatory elements in cancer are still an enigma. Here we hypothesize that across the cancer genome, sites that once had developed an enhancer chromatin state throughout the history of the cancer cells would be more prone to be activated and to stimulate gene transcription in cancer than sites that had never been enhancers. Thus, the repertoire of enhancers established during the developmental history of cancer cells may define their expression potential.

We and others have previously shown that the DNA methylation level is closely linked to enhancer activity states [25–28] and to expression levels of associated cancer genes [22,25]. We now utilized DNA methylation data to investigate the fate of ES enhancers during normal hematopoietic development and in cancer. Surprisingly, we discovered that enhancers present in ES cells and absent from differentiated hematopoietic cells are especially prone to DNA methylation alterations in cancer. Moreover, these changes in methylation are in accordance with the expression levels of adjacent genes, and thus may provide a basis for the reactivation of ES genes in cancer.

## Results

### DNA methylation characterizes enhancer dynamics during development of the hematopoietic cell lineage

**Fig 1:**
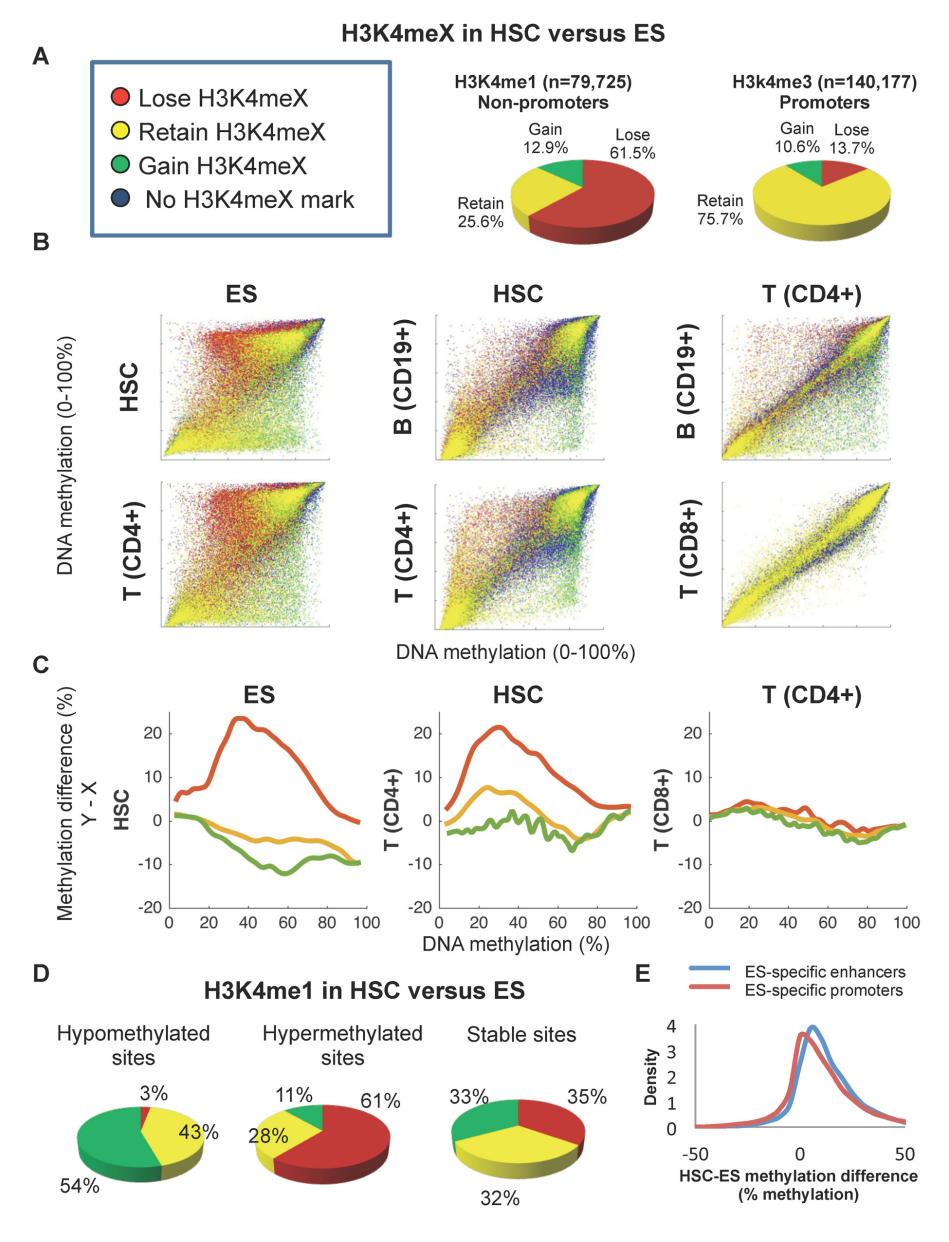
Developmental DNA methylation changes in regulatory sites. **A.** Fractions of methylation sites that lose, maintain, or gain H3K4 chromatin marks in hematopoietic (HSC) stem cells versus ES cells. **B.** Methylation levels of later (y axes) versus earlier (x axes) developmental stages for sites that lose (red), retain (yellow), or gain (green) H3K4me1 signals during differentiation. Methylation levels in T and B cells are given as well. **C.** Methylation differences between later to earlier stages as a function of the earlier methylation level, for sites that lose (red), retain (yellow), or gain (green) H3K4me1 signals. **D.** Sites that are hypo-, hyper- or similarly methylated in HSC compared with ES, categorized by H3K4me1 marking in HSC versus ES. **Left:** sites that became hypomethylated (at least 20% methylation loss). **Middle:** sites that became hypermethylated (at least 20% methylation gain). **Right:** sites that maintained similar methylation levels (less than 20% methylation difference). **E.** Distribution of DNA methylation differences between HSC and ES for ES-specific enhancers and promoters.

We explored the fate of ES enhancers during normal development of the hematopoietic cell lineage. DNA methylation and chromatin data relating to six human ES cell lines, seven hematopoietic stem cell (HSC) samples, and twelve T and B-cell derivatives of the hematopoietic lineage were downloaded from public databases (S1 Table). A total 179,910 non-promoter, non-CpG island methylation sites were located and the prevalence of the H3K4me1 chromatin mark typical of enhancers around these sites was analyzed (S1A Fig). Also, 229,306 promoter methylation sites were located and H3K4me3, a mark typical of gene promoters around these sites was analyzed (S2A Fig). Many sites showed a loss of H3K4me1 marks in HSC versus ES: out of 79,725 H3K4me1 sites, 49,031 (61.5%) lost the mark in HSC, whereas 20,380 (25.6%) maintained it in ES and only 10,314 (12.9%) gained it in the HSC. The tendency to lose H3K4me1 between ES to HSC was strictly associated with enhancers, as only 13.7% of the promoters with the H3K4me3 mark in ES showed a loss of the mark in the HSC (Fig 1A). Moreover, this massive loss of enhancer mark was particularly notable between ESs and HSCs, because in differentiated CD4^+^, CD8^+^ T cells or CD19^+^ B-cells, only 27.4%, 25.7%, and 37.8% of the HSC H3K4me1-marked sites, respectively, were lost (S1 Fig). We termed these promoter-distal elements, which carry the H3K4me1 mark in ES but not in hematopoietic cells, the ES-specific enhancers (ESSEs) of the hematopoietic cell lineage.

Clearly, DNA methylation describes the chromatin state of ES enhancers. We found that enhancers which lose H3K4me1 tend to increase DNA methylation during development, enhancers that gain H3K4me1 tend to decrease methylation, and enhancers that retain H3K4me1 levels show no significant change in their methylation levels (Figs 1B, 1C and S1B). In addition, most of the hypomethylated sites in HSC versus ES were sites that gained the H3K4me1 mark between ES and HSC, most of the hypermethylated sites lost this mark, and most of the stable sites maintained it (Fig 1D). These results are in line with previous reports showing that ES enhancers tend to gain DNA methylation levels and decrease or lose enhancer chromatin marks and activity upon cell differentiation [17]. We also observed an association between DNA methylation and chromatin status in promoter sites (S1C and S2 Figs), but the effect was weaker than in the enhancers (Fig 1E). Cumulatively, the results confirmed an association between DNA methylation levels and enhancer chromatin states during cell differentiation.

### DNA methylation fates of ESSEs in cancer

**Fig 2:**
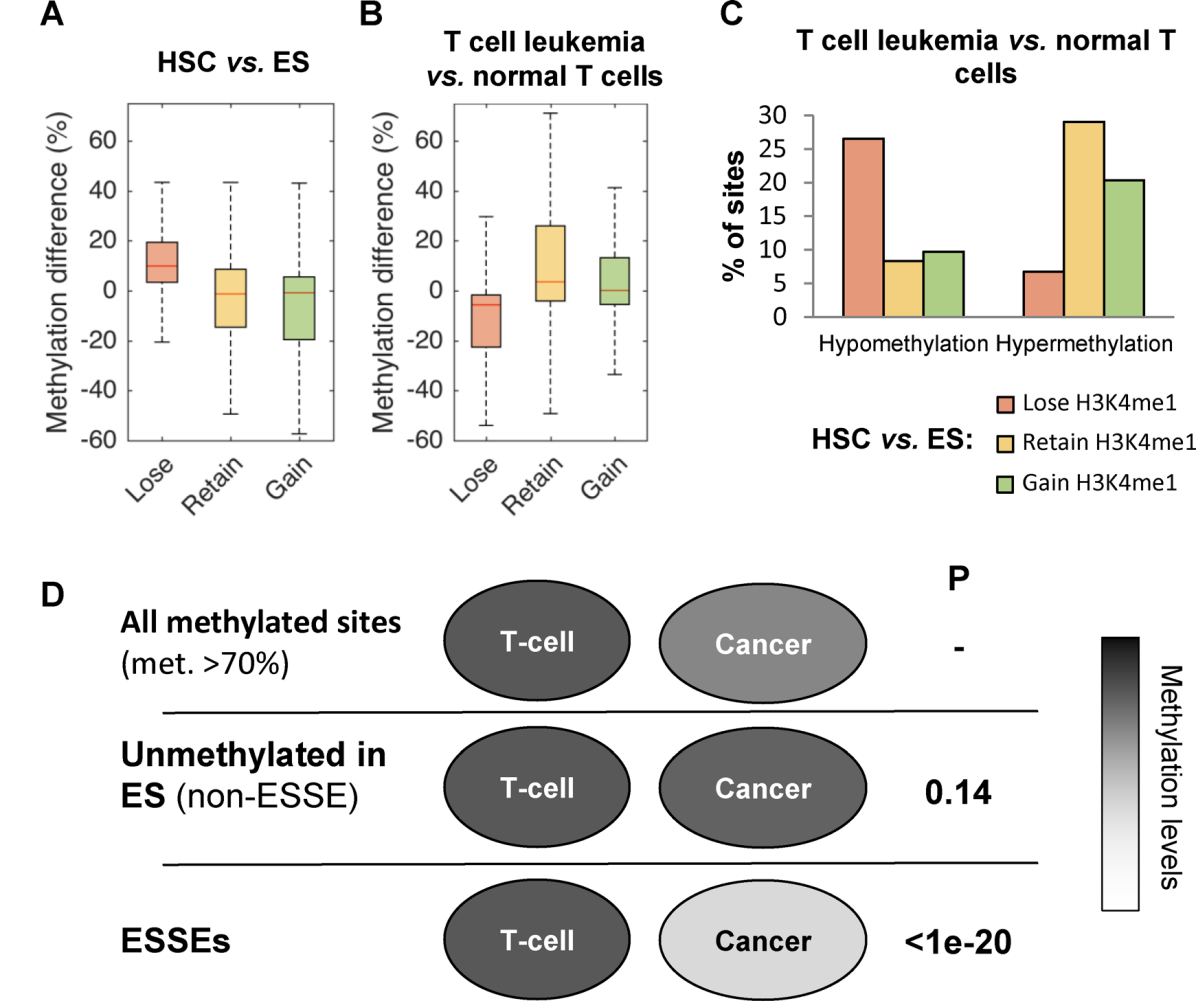
DNA methylation of ES-specific enhancers in cancer. **A.** Methylation differences between HSC and ES for enhancer sites that lose (red), retain (yellow), or gain (green) H3K4me1 in HSC compared to ES. **B.** Same analysis between T cell leukemia (Jurkat) and normal CD4^+^ T cells. **C.** Percentage of sites that were hypomethylated or hypermethylated by at least 20% in T cell leukemia compared to normal T cells. **D.** Schematic representation of the methylation fates in the cancer cells of sites that were highly methylated in normal T cells. P-values were calculated for the null hypothesis of no difference from the fate of T cell methylated sites in cancer.

We next explored the methylation status of ESSEs in acute T cell leukemia cells (Jurkat) versus that of normal mature CD4^+^ or CD8^+^ T cells. Strikingly, the ESSEs, which generally gained methylation in differentiated white blood cells compared with embryonic and tissue stem cells (Figs 1, 2A and S3A), were specifically prone to the loss of methylation in the cancer, compared with normal T cells (Fig 2B and 2C). On average, ESSE sites displayed 11.0% and 8.3% lower methylation levels in Jurkat cells than in normal CD4^+^ or CD8^+^ T cells, respectively (*p-value <* 1e-20). In contrast, constitutive (i.*e*. ES and HSC) and HSC-generated enhancers gained 10.9% and 5.1%, respectively, in Jurkat cells, versus that in normal CD4^+^ cells. Similar results were obtained for CD8^+^ T cells (S3B Fig).

Overall, 26.5% of the ESSEs were hypomethylated in cancer cells compared with normal T cells, whereas only 6.7% became hypermethylated (red bars in Fig 2C). In contrast, 20.3% and 29.0% of the retained and gained sites, respectively, became hypermethylated, whereas only 9.7% and 8.3% of these sites, respectively became hypomethylated (Fig 2C).

Moreover, this hypomethylation of the ESSEs in the cancer was not a mere reflection of their tendency to be hypomethylated in ES, as non-enhancer sites that were unmethylated in ES did not display this trend (Fig 2D and S2 Table). Thus, ES-specific enhancers display a unique tendency to become hypomethylated in cancer, a tendency not seen in constitutive or hematopoietic-specific enhancers.

### Polycomb-repressed genes harbor ESSEs that are hypermethylated in cancer

**Fig 3:**
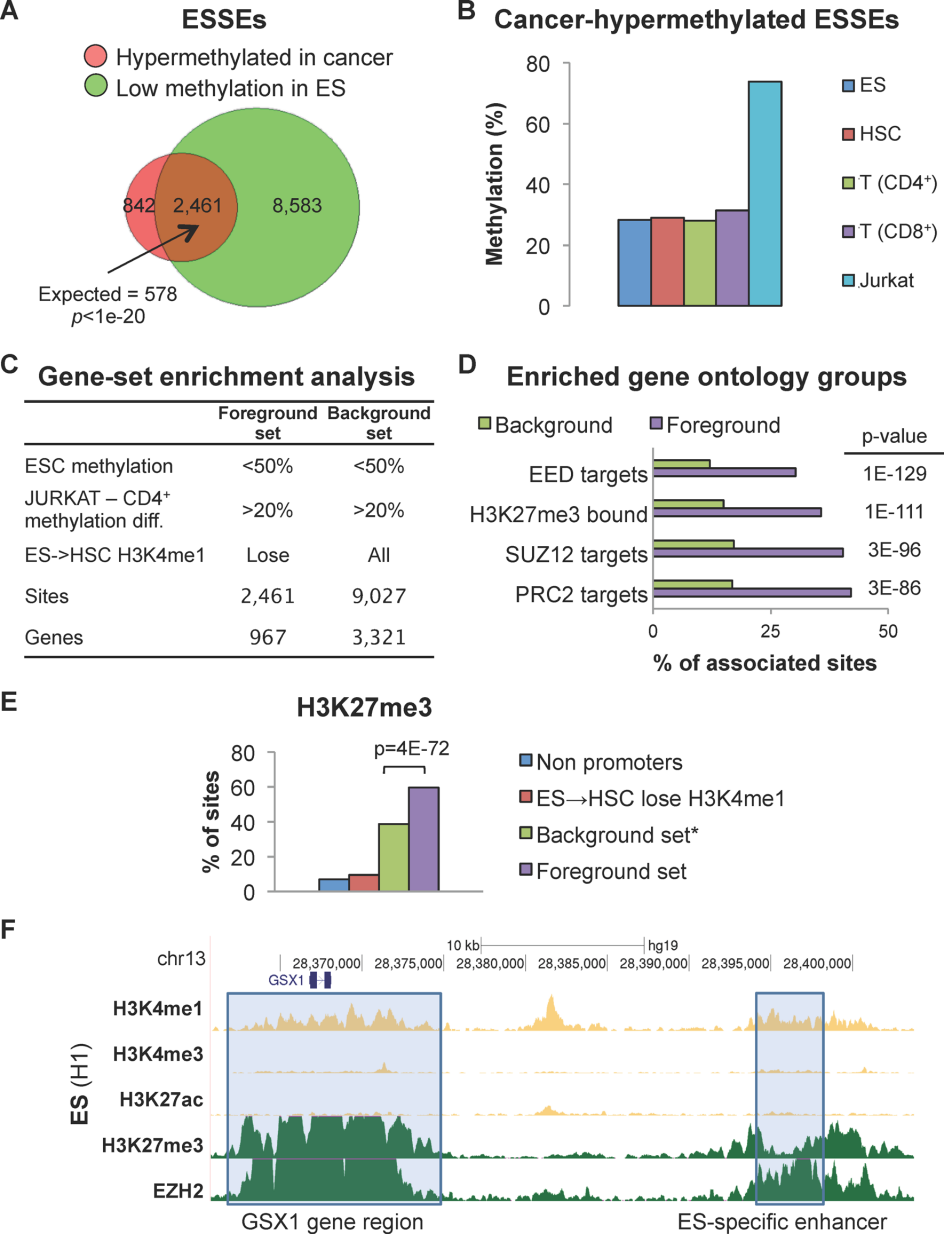
Polycomb-repressed genes have hypermethylated enhancers. **A.** Overlap between ESSE sites that gain methylation in cancer and ESSE sites that are undermethylated in ES. **B.** DNA methylation levels of ES-undermethylated-cancer-hypermethylated ESSEs during development. **C.** Foreground and background sets for the analyses shown in D and E. **D.** Gene set enrichment analysis of the gene ontology groups adjacent to sites in the background versus the foreground sets. The most significantly-enriched gene ontology groups proximal to hypermethylated ESSEs are indicated. **E.** Fractions of enhancer sites carrying H3K27me3 marks in ES. **F.** An example of cancer-hypermethylated ESSE in theGS Homeobox 1 (GSX1) gene region. Gray boxes indicate a region around the gene (left) and an ESSE region 25 kb upstream from the gene (right) marked by H3K27me3 and bound by the polycomb-related protein EZH2 in ES cells. These regions are hypermethylated in cancer.

We further characterized the hyper- and hypo-methylated ESSEs. First, we analyzed the small group of ESSEs that became hypermethylated in cancer. A set of ESSEs (n = 3,303) showed hypermethylation in cancer relative to normal T cells. The vast majority of these sites (74.5%, *p-value* < 1e-20) were undermethylated (methylation level ≤ 50%) in ES cells (Fig 3A). Despite the general tendency of ESSEs to become methylated in HSC, we found that this small, unique group of ESSE sites maintained low DNA methylation levels in ES, HSC, and differentiated T cells (Fig 3B). To explore whether these hypermethylated ESSEs displayed a common regulatory characterization, we analyzed their association with particular functional gene groups. Utilizing the GREAT platform [29], we scanned various gene-ontology databases, and compared the foreground set of genes, (*i*.*e*. the genes in the vicinity of the 2,461 ESSEs that were unmethylated in all the normal cells and hypermethylated in cancer), to a background set of the genes (i.e. genes near 9,027 enhancer sites, whether ESSE or not, that were hypermethylated in cancer) (Fig 3C). Strikingly, the analysis revealed that the hypermethylated ESSEs were significantly enriched (*p-value* = 3e-86) in the vicinity of genes whose promoters were bound by the polycomb group (PcG) proteins (Fig 3D and S1 Dataset). Thus, the hypermethylated ESSEs were specifically enriched around PRC2-targeted genes.

It has been shown that methylation sites in the promoters of polycomb-repressed genes maintain low methylation levels in ES cells and during development, but become hypermethylated in cancer [9,10]. A characteristic feature of these promoters is the bivalent H3K4me3-H3K27me3 chromatin signature in ES cells, but not necessarily in later developmental stages or in cancer. Nevertheless, their repressed state is maintained in cancer, presumably through DNA methylation [30]. Our analysis showed that the hypermethylated ESSEs were significantly associated (*p-value* = 1e-111) with genes with H3K27me3-bound promoters (Fig 3D). We then explored whether these ESSEs carry the H3K27me3 mark in ES cells as well. Indeed, in ES cells the foreground set of hypermethylated ESSEs was significantly enriched with the H3K27me3 mark, and hence by the bivalent H3K4me1-H3K27me3 chromatin signatures, compared with non-ESSEs (*i*.*e*., the background set) (*p-value* = 4e-72), to ESSEs that were not hypermethylated in the cancer cells, or to all non-promoter regulatory sites (Fig 3E). A typical example is shown in Fig 3F. Thus, enhancers adjacent to PcG-regulated genes carry the chromatin signatures typical of PcG-bound regulatory sites in ES cells. Similar to PcG-targeted promoters, these enhancers tend to be hypermethylated in cancer compared with normal tissue.

### Hypomethylated ESSEs

**Fig 4:**
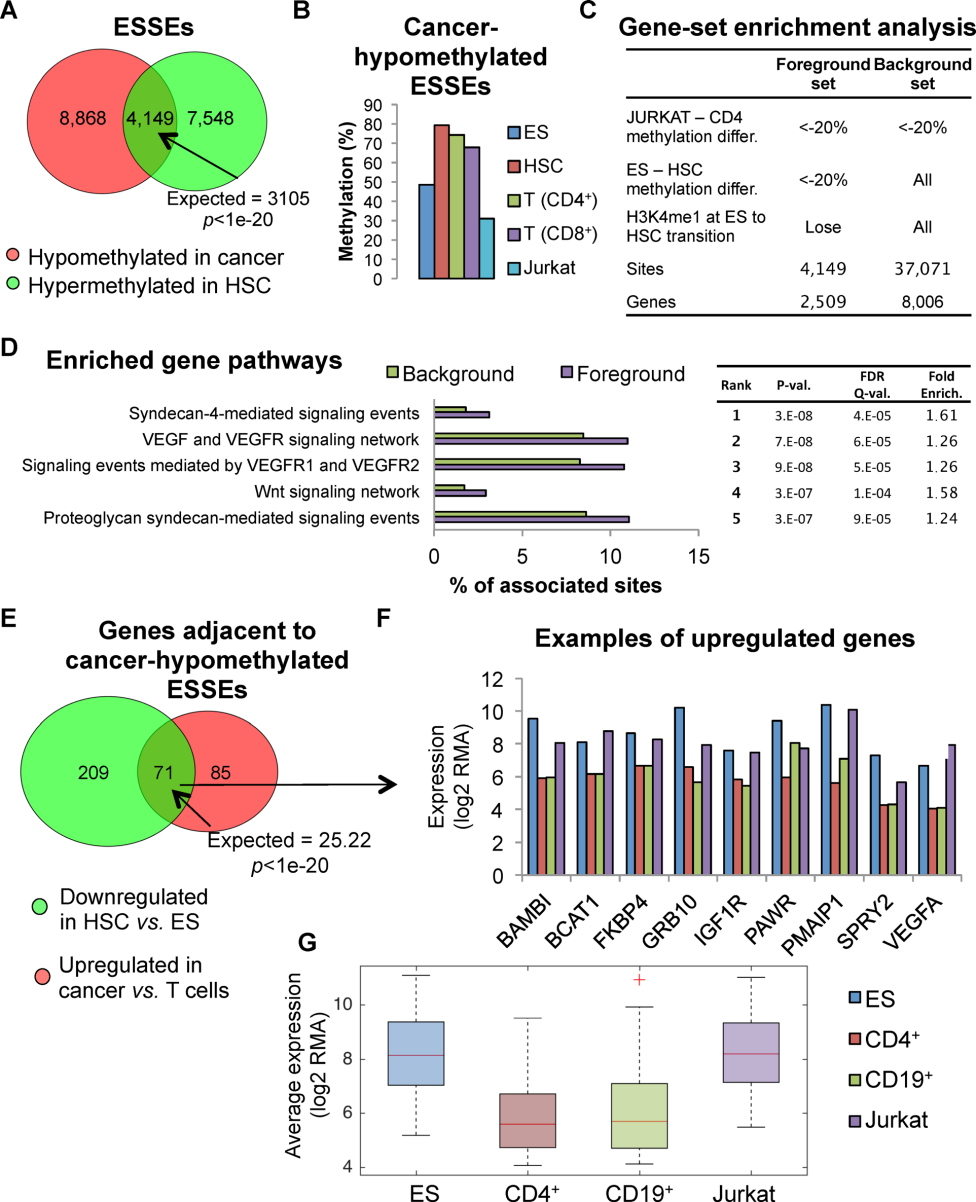
Cancer-hypomethylated ES specific enhancers. **A.** Overlap between ESSEs hypomethylated in cancer (compared with normal T cells) and ESSEs hypemethylated in HSC compared with ES. **B.** Methylation of cancer-hypomethylated ESSEs during development. **C.** Definition of foreground and background sets for the gene set enrichment analysis shown in D. **D.** Gene ontology enrichment analysis of background *versus* foreground sets. **E.** Overlap between genes adjacent to cancer-hypomethylated ESSEs that were down-regulated in HSC compared with ES or upregulated in cancer. **F.** Expression patterns of example genes next to hypomethylated ESSEs in ES, normal lymphocytes, and Jurkat leukemia cells. **G.** Distribution of the average expression levels of the reactivated genes shown in panel 4F.

As shown above, a large group of ESSEs (n = 13,017) displayed hypomethylation in leukemic cells compared with normal T cells (Fig 2C). A significant subgroup of these enhancers (n = 4,149) was hypermethylated in HSC compared with ESCs (*p<*1e-20) (Fig 4A). Thus, a considerable group of ESSEs was undermethylated in ES cells, methylated in HSC and T cells, and hypomethylated in cancer (Fig 4B). This hypomethylation was specific to ESSEs, as HSC-specific enhancers did not display this trend (S4 Fig). Gene set enrichment analysis of the genes in the vicinity of these hypomethylated-in-cancer ESSEs revealed a significant enrichment of developmental genes participating in VEGF, WNT, and other signaling pathways (Fig 4C and 4D, S3 Table). Moreover, genes proximal to cancer-hypomethylated ESSEs that were expressed in the ES cells but down-regulated in the hematopoietic linage significantly tended to be expressed in cancer (*p<*1e-20) (Fig 4E–4G). These results suggest an association between hypomethylation of ESSEs that are normally methylated in differentiated cells, and up-regulation of ES genes in cancer cells. Many of these genes participate in developmental signaling pathways.

### A hypomethylated-in-cancer ESSE supports VEGFA expression

**Fig 5:**
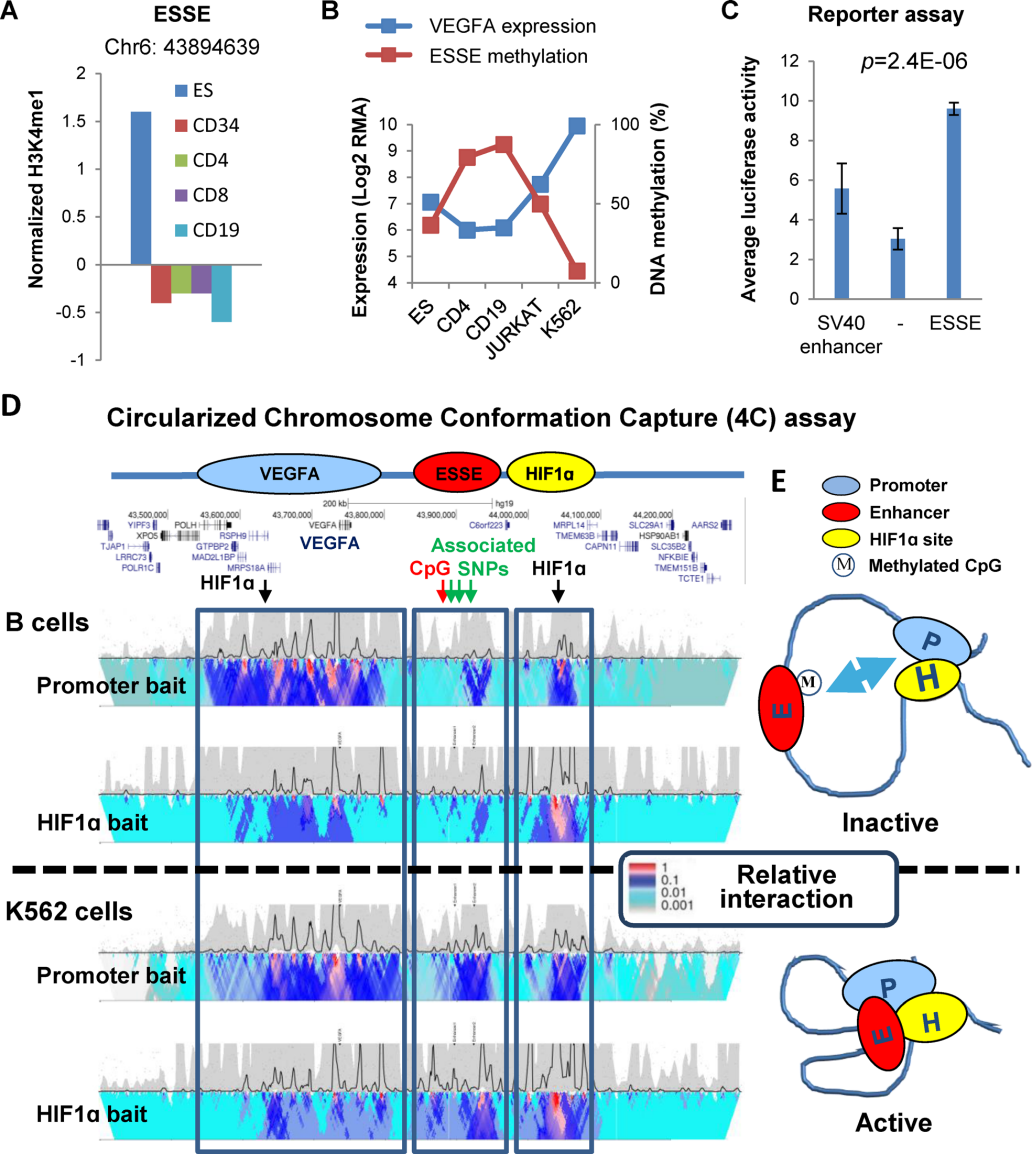
A hypomethylated ESSE regulates VEGFA expression. **A.** H3K4me1 mark of an ESSE site 156.7 kb downstream from the VEGFA gene promoter in ES, HSC (CD34^+^), T (CD4^+^, CD8^+^), and B (CD19^+^) cells. **B.** Expression levels of the VEGFA gene and methylation (%) of the downstream ESSE site in ES, T, B, and acute (Jurkat) and chronic (K562) leukemia cells. **C.** Relative expression level of a luciferase reporter gene from a minimal SV40 promoter with the SV40 enhancer (left bar), no enhancer element (middle), or a 1.5 kb DNA segment surrounding the ESSE site, in K562 cells. Error bars indicate the results of 5 independent experiments, 4–7 technical repeats each. The P value denotes the expression difference between the minimal promoter alone and the promoter with the ESSE element. **D.** Circularized Chromosome Conformation Capture and sequencing (4C-seq.) analysis of the VEGFA promoter (promoter baits) and of HIF1-alpha binding site (HIF1ɑ baits) interactions in B-lymphocytes (GM12093 line) and in K562 cells. Color code indicates the relative number of junction sequence reads between the promoter and the surrounding sequences. The Y-axis denotes relative enrichment of signals in smaller (top) and larger (bottom) windows across the region. The locations of genes, HIF1-alpha binding sites, and the correlated CpG methylation site are marked above. No other associated regions appeared across the genome, and no other HIF1-alpha binding site appeared in 10 Mb around VEGFA. **E.** Model of regulatory long-distance chromatin loops around the VEGFA promoter (P): in addition to constant interaction with a HIF1-alpha region, VEGFA-expressing cells also display enhanced interaction with the ESSE site in its unmethylated form.

We hypothesized that hypomethylated ESSEs are important regulators of reactivation of ES gene expression in cancer. To explore this hypothesis, a hypomethylated ESSE site located 156.7 kb downstream to the VEGFA gene was selected for in-depth analysis (Fig 5A). Through a set of experiments in living leukemic cells we explored whether this ESSE regulates VEGFA expression. This site was chosen as the role of VEGFA in various cancer types, including leukemia, is well documented [31–33]. However, the mechanism underlying VEGFA activation in cancers is unknown. Furthermore, VEGFA is a main component in the enriched network of genes located in proximity to hypomethylated ESSEs (Fig 4D). VEGFA was strongly expressed in ES cells, significantly downregulated in B and T lymphocytes, and reactivated to the ES level in T cell leukemia (Fig 4F). In accordance, the methylation of the linked ESSE site was negatively correlated with VEGFA expression in ES, normal T and B cells, T cell leukemia (Jurkat) and erythroleukemia (K562) cells (Fig 5B). To determine whether this ESSE is able to enhance transcription, a 1,500 bp fragment surrounding the ESSE methylation site was cloned upstream to a minimal SV40 promoter in a luciferase reporter plasmid and transfected to K562 cells. Interestingly, this ESSE significantly enhanced expression of the reporter gene (*p* = 2.4E-6) (Fig 5C). An important characteristic of enhancers is their ability to support transcription regardless of their orientation relative to the promoter region. Indeed, the ESSE segment significantly enhanced expression when placed upstream or downstream to the minimal promoter. Thus, the DNA element surrounding the ESSE site is a transcriptional enhancer in leukemia cells.

We further investigated whether the predicted ESSE site controls the expression of the VEGFA gene at its endogenous locus. First, we explored the physical interaction of the predicted sites with the VEGFA promoter through the long-range chromatin loop, a characteristic mark of enhancers [34]. The Circularized Chromosome Conformation Capture (4C-seqencing) assay allowed mapping the interactions between the VEGFA promoter and DNA sequences across the K562 cell genome. Across the entire genome only three genomic regions interacted with the VEGFA promoter: a large region surrounding the VEGFA gene (expected from this type of analysis); a region harboring the ESSE site located about 160 kb downstream from the promoter; and a region about 300 kb downstream from the gene (Fig 5D). Interestingly, the ESSE-containing region also contains single nucleotide polymorphisms (SNPs rs9472155; rs11755845; rs4513773) previously associated with the circulating VEGFA level, whereas the more downstream region binds HIF1-alpha, a key regulator of VEGFA expression in human cells (hg19-chr6:44,040,242–44,045,241; HepG2 GSE16347; HUVEC GSE39089). Of note, in addition to the above described HIF1-alpha site, there is only one more HIF1-alpha binding site, located 5’ to the VEGF promoter, in the 10 Mb surrounding the VEGFA gene. Thus, we will refer to the region located 300 kb downstream from the VEGFA gene and harboring the HIF1-alpha binding site as the HIF1-alpha region. As shown, the association between the VEGFA promoter and the HIF1-alpha binding region appeared constitutive in both normal B and leukemic cells, whereas the interactions of both regions with the ESSE-containing region were significantly stronger (*p-value* = 1.1E-14) in cancer (Fig 5D). Thus, the VEGFA promoter and the HIF1-alpha binding region physically interact in both expressing and non-expressing cells, but the interaction of the two with the ESSE region, containing the genetic (SNPs) and epigenetic (methylation) polymorphisms, is significantly more notable in VEGFA-expressing cells and when the ESSE is less methylated (Fig 5E).

**Fig 6:**
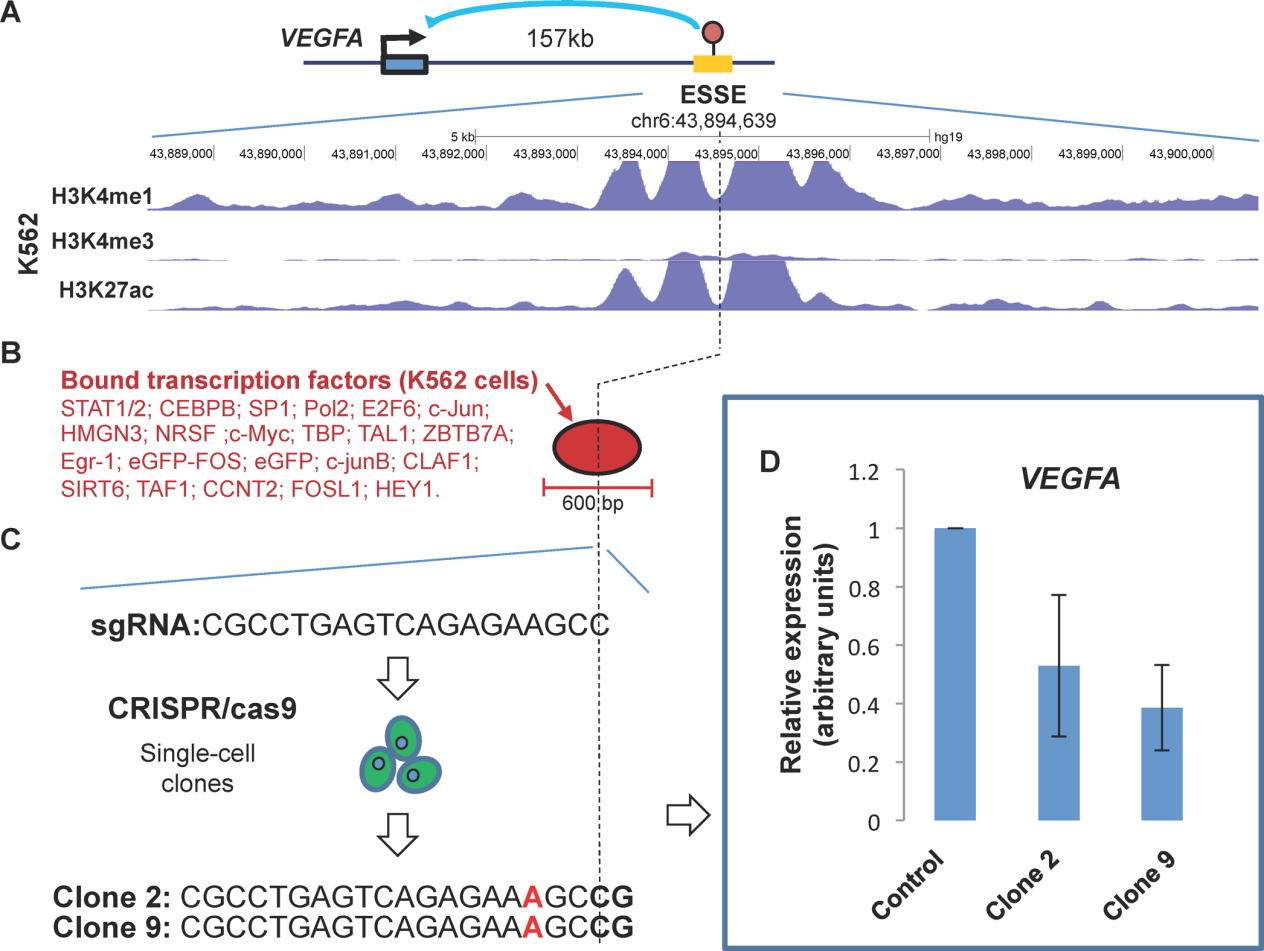
Validating a predicted ESSE as a novel VEGFA enhancer. **A.** Genomic map of the chromatin state around the predicted ESSE site in K562 cells showing the typical chromatin signature (H3K4me1/H3K27ac) of active enhancers. The location of a VEGFA-associated SNP (rs9472155) is indicated. The location of the correlative CpG methylation site is marked by a vertical dashed line. **B.** Focused (<600 bp) cluster of transcription factor binding sites (Chip-seq data) within the predicted enhancer. The factors bound in K562 cells are listed. This is the only cluster within 20 kb around the ESSE. **C.** Sequence of the guide RNA used to target the Cas9 endonuclease at the correlative methylation site, and the resulting mutation (insertion) in two independently-analyzed single-cell clones of the transfected K562 cells. **D.** Expression levels of the endogenous VEGFA gene in the mutated clones relative to mock-treated cell. Results of three independent qPCR experiments with three technical replications of each experiment are shown.

To determine whether VEGFA expression depends upon the ESSE, we mutated the ESSE site in K562 cells by utilization of the CRISPR/Cas9 genome-editing system. Strikingly, the introduction of a point mutation only 2 bp apart from the ESSE methylation site, within a cluster of transcription factor binding sites, significantly reduced VEGFA expression (Fig 6). Thus, this site not only correlates with VEGFA expression, but plays a causative role in the reactivation of VEGFA in the blood cancer cells.

**Fig 7:**
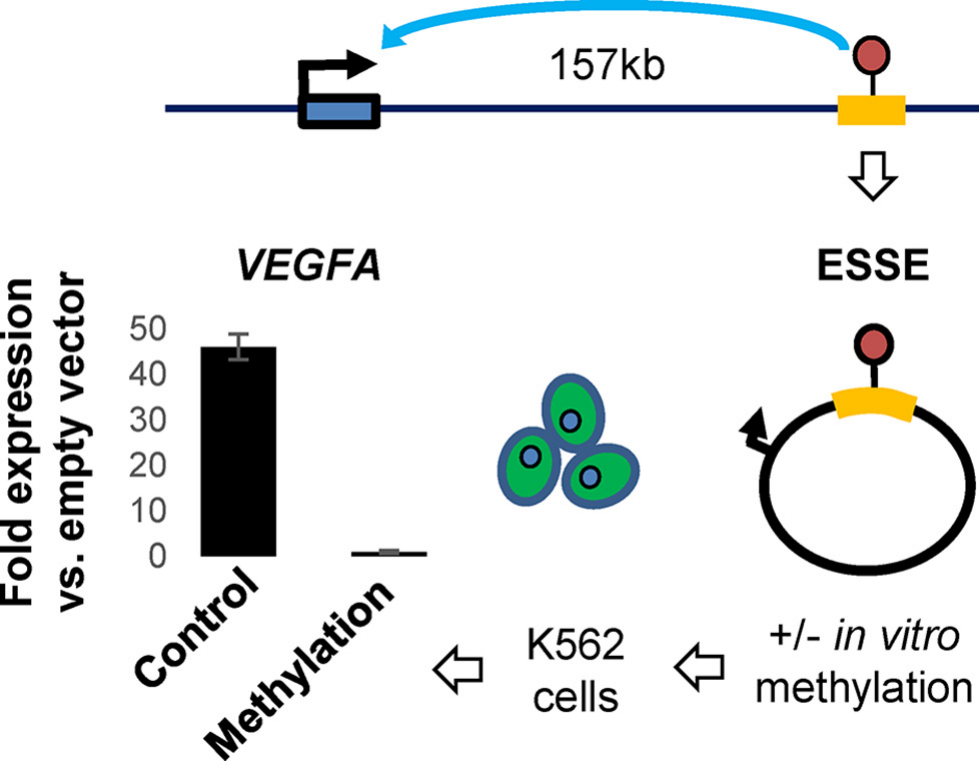
*In-vitro* methylation experiments. The 1.5 kb segment surrounding the ESSE site was integrated downstream to the minimal promoter of the luciferase reporter gene and introduced to K562 cells before or after *in-vitro* DNA methylation. Relative luciferase activity compared with the empty unmethylated or methylated vector is shown.

Finally, we explored the effect of methylation on the *in vivo* enhancer activity of the ESSE. With the aid of an *in vitro* methylation assay, we methylated the reporter vector, containing the ESSE element upstream or downstream of minimal promoter, and evaluated the luciferase activity compared with that of the unmethylated vector in K562 cells. Clearly, the results showed that DNA methylation significantly reduced the ability of the ESSE to support transcription (Fig 7).

Cumulatively, these findings show that the ESSE, initially predicted based on its correlation with the chromatin state, is a distal regulatory element that controls VEGFA expression in a methylation-dependent manner.

### Across- tumor ESSE methylation

**Fig 8:**
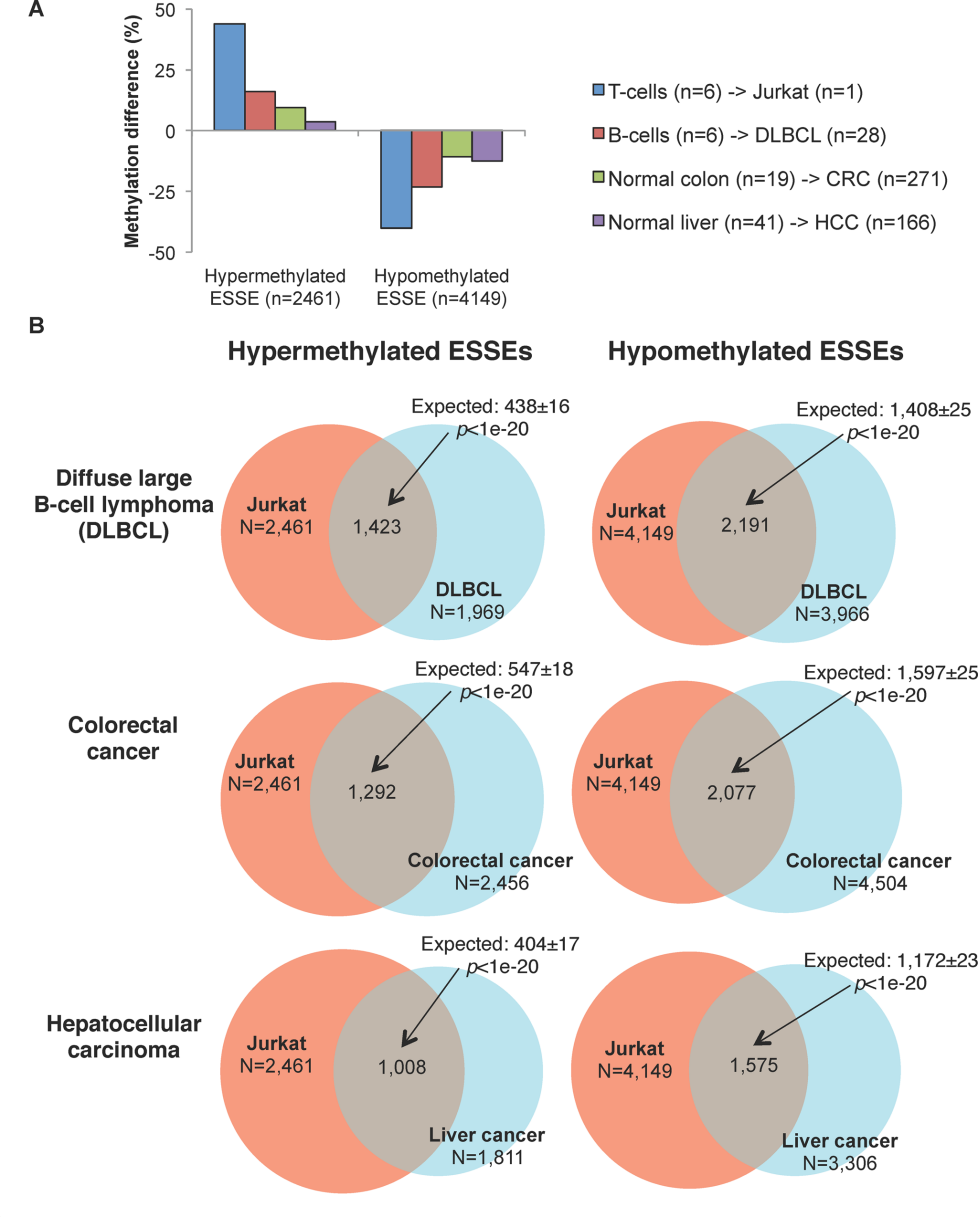
Methylation trends of ESSEs in fresh cancer samples. **A.** Average cancer-normal methylation differences of the ESSEs that were differentially methylated between Jurkat and normal T cells, in diffuse large B-cell lymphoma (DLBC), colorectal cancer (CRC), or hepatocellular carcinoma (HCC). The number of fresh tumor samples is indicated. **B.** Overlay of hyper- and hypomethylated ESSEs in Jurkat and other cancers.

To investigate whether the effects we observed in the lymphoma cell lines also existed in fresh tumor samples, we analyzed the overlap between hyper- and hypomethylated ESSEs in Jurkat cells compared with that in diffuse large B-cell lymphoma (DLBCL) samples (n = 28) obtained from the TCGA project. The ESSE sites that were hypermethylated in the Jurkat cells were also preferentially hypermethylated in the lymphoma samples, and the hypomethylated ESSEs maintained hypomethylation in the lymphomas, albeit not to the same extent as in the cell line (Fig 8A). Moreover, the overlap between the hyper- and hypo-methylated ESSEs in the Jurkat cells and DLBCL samples was highly significant (*p-value <* 1E-20) (top panels, Fig 8B). Thus, in the lymphoma lines and in the fresh samples, the same sites displayed the same trends.

We also analyzed ESSE methylation drifts in non-hematopoietic cancers. The analysis revealed a highly significant overlay (*p-value <* 1E-20) between hyper- and hypo-methylated ESSEs in lymphoma, colorectal cancers (CRC) and hepatocellular carcinomas (HCC) (Fig 8B). It appears that cancers of various tissues of origin apparently share the methylation trends of ESSEs.

### Methylation of the VEGFA enhancer predicts VEGFA expression across cancers and patients

**Fig 9:**
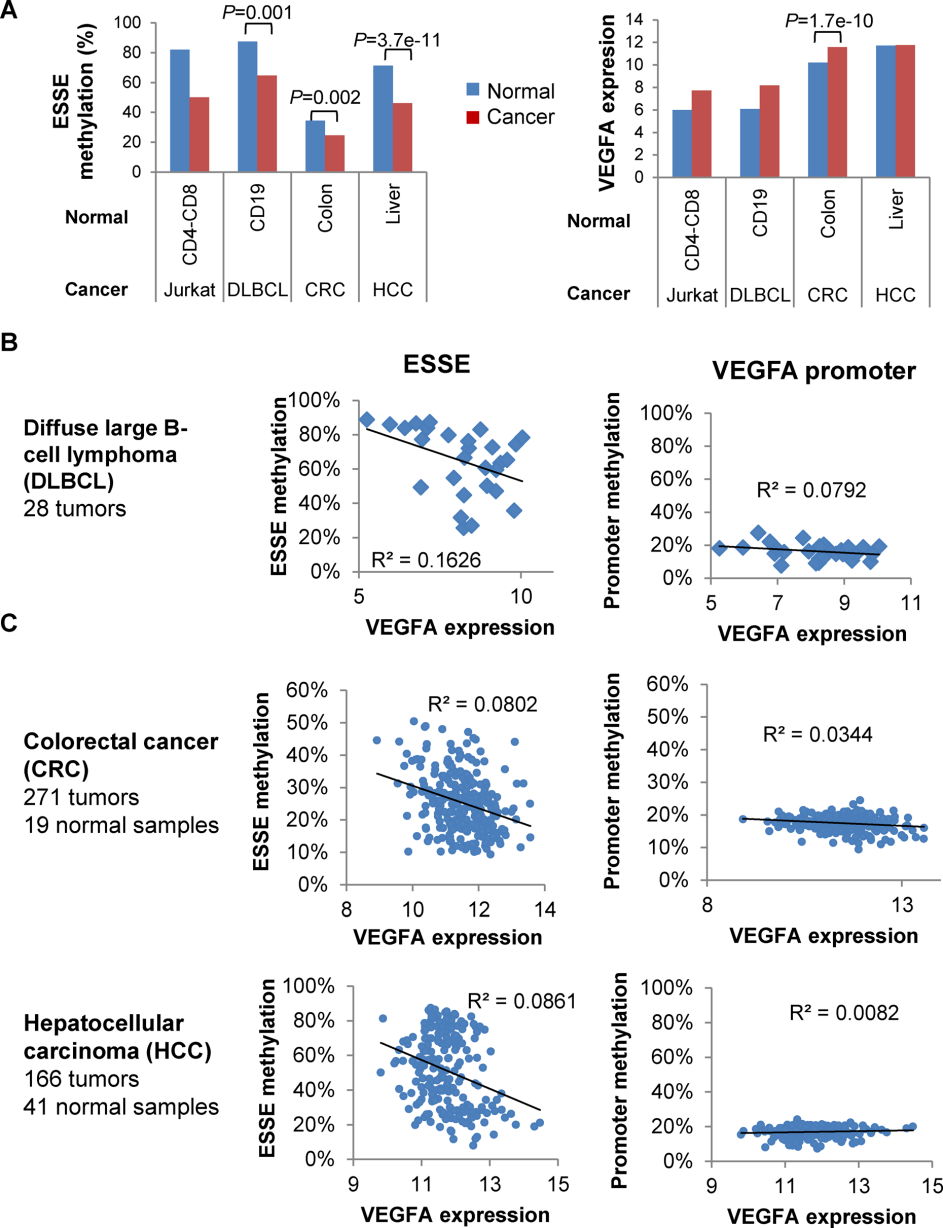
ESSE methylation and across-patient VEGFA expression variation. **A. Left:** Average methylation of VEGF ESSE in cancer and normal samples. P-values of the cancer-normal differences are indicated for analyses including more than one normal and cancer sample. **Right:** Same analyses for VEGF expression level. **B. Left:** Correlation between methylation of VEGFA ESSE and VEGFA expression levels across DLBC patients. **Right:** Correlation between methylation of the VEGFA promoter (average of nine methylation sites within 1 kb of TSS) and VEGFA expression levels across DLBC patients. **C.** Same analyses as in B for normal and cancerous colon and liver samples.

We explored the relationships between methylation of the VEGFA enhancer site and expression of the VEGFA oncogene across cancer types and patients. We found that the VEGFA enhancer was significantly hypomethylated in all the examined cancer types (Fig 9A, left). Interestingly, this gene was upregulated in DLBCL and CRC compared with that in the corresponding normal tissues, whereas in normal liver its level of expression was as high as in HCC (Fig 9A, right). This may indicate that the ESSE methylation status is not a mere manifestation of the gene expression status or an absolute requirement for gene expression, but rather a cancer-related process which may support reactivation of silenced genes. To further understand these associations we analyzed the VEGFA expression-methylation relationships across patients. We found a considerably large variation in the mRNA expression level of this gene across normal tissues and DLBCL, CRC and HCC tumors. Strikingly, methylation of the ESSE site was well correlated with VEGFA expression in the examined cancers. In contrast to the correlation described for enhancers, and in accordance with our previous analyses [25], the association between promoter methylation and gene expression level was poor and in DLBCL and HCC not statistically significant (Fig 9B and 9C). Cumulatively, the results indicate that ES enhancers are specifically prone to methylation perturbations in cancers. These alterations are associated with the gene expression potential of tissues, individuals and diseases.

## Discussion

Many tumors display mixtures of co-expressed lineage-specific and stem cell genes. This has led to the assumptions that tumors originate in undifferentiated cells embedded in the tissue, from dedifferentiation of tissue cells induced by carcinogenetic signals, or from activation of particular stem cell genes through stochastic regulatory mutations later selected by the developing cancer. Our results support an alternative scenario, in which upon loss of control over the global methylome typical of many cancer types, sites that already carry particular regulatory chromatin structures are prone to specific silencing or reactivating methylation modifications. In turn, these modifications may alter gene expression. Among gene regulatory sites, enhancers are particularly versatile owing to their tissue-specific chromatin structures. Indeed, several studies have shown that reprogramming of enhancer activity during cellular transformation such as acquisition of a drug resistance phenotype and other cancer-specific characteristics [35,36] plays an essential role in cancer development and progression [22–24]. However, little is known about the mechanisms that underlie epigenetic rewiring. Our results indicate that patterning of enhancer chromatin along the developmental history of cells provides frameworks for gene activity from which cancers may further select their expression repertoire.

The first important observation is that enhancers that appear only at the very beginning of the hematopoietic cell lineage commitment are specifically prone to methylation perturbation in blood cancers versus gene promoters and enhancers that are generated in blood stem or differentiated cells (Figs 1 and 2). These results are in line with recently published data showing that ES-specific accessible chromatin elements are target for epigenetic reprograming during cancer development, as identified by DNase I hypersensitive site mapping [8].

Second, we show that the level of DNA methylation characterizes the activity state of enhancers and associated genes (Figs 3, 4, 5 and 9), consistent with previous reports from our and other research groups in other model systems [22,25],[37,38]. The mechanism underlying this association is unclear, and may involve active adaptation of the DNA methylation level upon enhancer specifications, the effect of methylation on transcription factor binding, and possible feedbacks from the gene transcription machinery. In addition, genes are frequently controlled by sets of enhancers, which may be affected by various activity factors. Therefore, unmethylated enhancers do not necessarily dictate gene activity and methylated enhancers do not automatically eliminate gene expression. In line with these notions, our findings show that enhancer hypomethylation is not a simple outcome of the gene expression status or an absolute requirement for gene expression (Fig 9). Rather, we suggest that ES-specific enhancers provide a framework for gene activity profiles upon global methylation perturbation in cancer.

Indeed, our genomic analyses of open resource data revealed a highly significant association between the methylation state of the predicted ESSE sites and the activity state of the corresponding genes in ES and cancers. To reveal whether these analyses identify actual regulatory sites that affect the expression potential of ES genes in cancer, we further performed a set of validating experiments. As a model gene we chose VEGFA, an important cancer gene that is expressed in many types of cancer, including lymphomas, and in ES cells, but not in differentiated white blood cells. Beyond the significant co-localization of ESSEs with the VEGF-A signaling pathway genes, including VEGFA itself (Fig 4A–4D), and the tendency of these genes to be upregulated in the cancer (Fig 4E and 4F), we also characterized a particular site predicted to be a novel VEGFA enhancer. We show that the predicted site displays the full list of essential enhancer characteristics, such as the ability to drive expression from a minimal promoter in the relevant cell type when placed upstream or downstream to the gene, and the physical interaction with the gene (Fig 5). Moreover, the predicted site does not only interact, but plays a causative role in controlling VEGFA activity in the cancer, as shown by the genome-editing experiments (Fig 6). Finally, we provide experimental evidence that the DNA methylation state of this ESSE site strongly affects its activity level as a transcriptional enhancer in leukemia cells (Fig 7). Cumulatively, these experiments strongly support our suggestion that the predicted sites, initially recognized through genomic correlation analyses, are *cis*-acting regulators that support the reactivation of ES genes in cancer.

Because ES enhancers are present at the origin of all cell types, they are expected to affect gene expression in various types of cancer. Our pan-cancer analyses (Figs 8 and 9) support this assumption. Interestingly, HSC enhancers were not prone to hypomethylation in hematopoietic cancers (Figs 2B, 2C and S4). This may indicate that enhancers of a given cell lineage are relatively immune to the global hypomethylation of cancer cells. This could be due to lineage-specific chromatin structures, which help to maintain an appropriate chromatin activity state in the face of the disturbed epigenome of cancer cells. ESSEs may lack these additional structures found in differentiated cells, and are thus more prone to the global methylation perturbations typical of cancer cells.

In conclusion, we reveal a novel pattern of regulatory epigenetic mutations in cancer. The new pattern is significantly more effective in ESSEs than in non ES-specific enhancers and promoters. Whereas the most reported pattern in gene promoters is hypermethylation of already-repressed genes, namely polycomb-repressed genes (with the exception of differentially methylated valleys (DMVs) in CRC) [4], the new pattern involves both hyper- and hypomethylations. This new pattern could participate in silencing particular ES genes in cancer, as well as lead to reactivation of ES genes that are silenced in normal somatic cells. The new pattern appears in both cancer cell lines and fresh tumor samples, and may predict the expression levels of important oncogenes across cancers and patients, as demonstrated for the VEGFA gene. The ability to detect this program with the limited genomic coverage provided by the available methylation methods highlights the potential for the discovery of additional key regulatory circuits in normal and cancer cells, once appropriate enhancer mapping methods have been developed.

## Materials and Methods

### Datasets

#### Histone modifications

The ChIP-seq data were downloaded from the National Institute of Health (NIH) Roadmap Epigenomics Mapping Consortium (http://www.roadmapepigenomics.org/) in WIG format aligned to build GRCh36/hg18. The positions were then lifted over to build GRCh37/hg19 of the human genome.

#### DNA methylation

Beta values of DNA methylation data created by the Illumina HumanMethylation450 BeadChip were obtained as follow: ES lines HUES9 (GSM1004633) and WA09 (GSM1004646, GSM1004647): the National Center for Biotechnology Information Gene Expression Omnibus (NCBI GEO); Jurkat cancer cell line: the ENCODE project; Purified CD34^+^ HSCs, CD4^+^ and CD8^+^ T cells, CD19^+^ B-cells: NCBI GEO (GSE35069). Level 3 Illumina HumanMethylation450 BeadChip of cancer and normal samples for diffuse large B-cell lymphoma (DLBC), colorectal adenocarcinoma (COAD) and liver hepatocellular carcinoma (LIHC) were obtained from the Cancer Genome Atlas (TCGA) (https://tcgadata.nci.nih.gov/tcga/dataAccessMatrix.htm).

#### Gene expression

Affymetrix Human Genome U133A Array CEL files for ESs, T and B cell samples, and Jurkat and K562 lines were downloaded from the NCBI GEO (S1 Table) and processed with the Affymetrix Expression Console with application of Multi-array Average (RMA) analysis. Level 3 RNA-seq data (RNAseqV2 normalized RSEM) were downloaded from the TCGA data portal (https://tcgadata.nci.nih.gov/tcga/dataAccessMatrix.htm) for normal and cancer samples of diffuse large B-cell lymphoma (DLBCL), colorectal cancer (CRC) and liver hepatocellular carcinoma (HCC).

#### Data processing

A methylation site was considered a promoter site if it resided within 2,500 bp of one of the 41,431 Transcription Start Sites (TSSs) in RefSeq. Non-promoter CpG sites were at least 5,000 bp away from TSSs, and not included within a CpG island site according to the sequence criteria in Gardiner-Garden & Frommer [24]. Histone modification levels were normalized to mean 0 and standard deviation 1. For each of the 482,421 CpG methylation sites on the Illumina 450 BeadChip array, the histone modification state was defined as the maximum normalized score within +/-100 bp of the methylation site. A CpG site was considered positive for a given modification if its average normalized level was >0. Gene set enrichment analyses for genes adjacent to ESSE sites were performed by MSigDB perturbation analysis with the use of the Genomic Regions Enrichment of Annotations Tool (GREAT) [29].

### Statistical analyses

All statistical analyses were performed with MATLAB. P-values, unless otherwise indicated, were calculated by the Wilcoxon rank-sum test for differences between averages. Distribution plots were smoothed using kernel smoothing density estimation. P-values of the overlay between sites (Venn diagrams) were calculated based on the expected chance intersections between the groups.

#### Luciferase reporter assay

A DNA fragment containing the ESSE site was amplified from K562 genomic DNA with the use of 5'GGAGAAACGCGTTGAGCAGGATAGTGGCATGA3' 5'GGAGAACTCGAGCCCAGCAACCCTTTTAGATC3' primers containing MluI and XhoI restriction sites, respectively, and cloned into a pGL3- reporter vector (Promega) upstream to a minimal SV40 promoter and a firefly luciferase reporter gene. For downstream cloning we used the above ESSE pGL3 vector as a template for primers 5' GTAGAATCTAGATTGAGCAGGATAGTGGCATG and 5'- GAGAAggccggccCTCGAGCCCAGCAACCCTTTTAG. The generated PCR product was inserted into pGL3 that had been digested with XbaI and FseI. Next, 5x10^5^ K562 leukemia cells were co-transfected with 1.8 mg of empty pGL3 vector, a control vector containing the SV40 enhancer, or the ESSE vectors, together with 130 ng of renila expressing plasmid, with the use of an Amaxa Nucleofector device. Cells were maintained in culture for 48 hrs. Firefly and Renilla luciferase intensities were measured in 8 replicates for each transfected cell group. Renila luciferase levels were used as the normalization factor for transfection efficiency and cell number, as described (34).The P value was calculated according to a 2-tailed T test of values from three independent experiments.

### Methylation experiments

The ESSE site was generated from the ESSE pGL3 reporter vector with the use of PCR primers 5'GTAGAAGGTACCTTGAGCAGGATAGTGGCATG and 5'GAGAAGAGCTCCTCGAGCCCAGCAACCCTTTTAG. The PCR product was inserted into pGL4.23 reporter vector (luc2/minP; Promega) upstream or downstream to the minimal promoter and the Firefly luciferase reporter gene after digestion with Acc65I and SacI. Complete in vitro CpG methylation of the ESSE pGL4.23 vector was performed with CpG Methyltransferase (M.SssI; Thermo Scientific), which was protected from HpaII (Thermo Scientific) digestion. A total 5x10^5^ K562 leukemia cells were co-transfected with 1.8 mg of empty pGL4.23 vector, ESSE pGL4.23 vector, methylated empty pGL4.23 vector or methylated ESSE pGL4.23 vector together with 130 ng of renila expressing plasmid.

### Genome editing experiments

#### Plasmid preparation

px330 vector [39] expressing Cas9 and sgRNA was digested with restriction enzyme BbsI and the linearized vector was gel purified. A pair of phosphorylated oligos targeting the ESSE methylation site were annealed and ligated to the linearized vector with the aid of a Rapid DNA Ligation Kit (Thermo scientific). The oligos sequences are: 5’CACCGCGCCTGAGTCAGAGAAGCC, 5’AAACGGCTTCTCTGACTCAGGCGC.

#### Cell transfection

K562 cells were maintained in RPMI 1640 supplemented with 10% FBS, 2mM L-glutamin, 1mM sodium pyruvate and 1% penicillin-streptomycin. The cells were transfected with 3.6 microgram of px330 vector containing the oligos mentioned above and 0.4 microgram of GFP-expressing vector by amaxa nucleofector 2b. Control cells were transfected with px330 without gRNA and with GFP-expressing vector with the same amounts as the cells in the experiment. After 24hrs 2μg/mL puromycin was added to the medium. The medium was replaced after 3 days with fresh medium without puromycin. GFP positive cells were isolated as single cells by FACS.

#### DNA extraction and sequencing

Genomic DNA was extracted (DNeasy, Qiagen) from each clone, according to the manufacturer's protocol. For PCR, we used the following primers in order to amplify the region with the mutations: forward primer 5’CCATCACTGCTCCACAATCA, reverse primer 5’ACTCCGAGTGGCTCCTAGTG. The amplified product was cloned into the pGEM-T vector (Promega Corporation, Madison, WI) and sequenced with SP6 primer.

#### Real-time PCR

Total RNA was isolated from the cells with the use of Tri reagent (Bio-lab). Reverse transcription was carried out with a Verso cDNA Synthesis Kit (Thermo scientific). The resulting cDNA was used as a template for RT PCR, which was performed with the Mx3005P System and MxPro QPCR software (Stratagene). Maxima SYBR Green/ROX qPCR Master mix (Thermo scientific) was used to perform PCR. PCR cycling conditions included a 95°C heating step of 10 min at the beginning of every run. The samples were then cycled 40 times at 95°C for 30 sec, 59°C for 20 secs, and 72°C for 30 sec. The plates were read after each cycle. A melting curve was generated at the end of the procedure to ensure product uniformity. Hypoxanthine guanine phosphoribosyl transferase (HPRT) was used as a housekeeping gene to compensate for between sample differences in in the amount of cDNA. The following primers were used: VEGF forward primer 5’CTACCTCCACCATGCCAAGT, VEGF reverse primer 5’GCAGTAGCTGCGCTGATAGA, HPRT forward primer 5’ TGACACTGGCAAAACAATGCA, HPRT reverse primer 5’GGTCCTTTTCACCAGCAAGCT. All samples were amplified in triplicate and the data was analyzed with the use of MxPro qPCR system software (Stratagene). Fig 7 show the average and the standard deviation of three experiments with three technical repeats each, relative to the control cells.

### Circularized Chromosome Conformation Capture (4C) assay

2×10^7 GM12093 or K562 cells were harvested from an exponentially growing culture and cross-linked with 1% formaldehyde, lysed and cut with the restriction enzyme DpnII (NEB). After ligation, reverse cross-linking, purification and second digestion with the restriction enzyme Csp6I (NEB), the DNA was circularized to obtain a 4C library, as described [36]. To achieve sequence junctions with the VEGFA promoter region, circularized DNA molecules were amplified by the 5'CCCCCTTTCCTGACTGGATC; 5'AAAGCTGTATCTGCCCTTTT primer set and sequenced on the Illumina MiSeq Sequencing System. Overall, 744,335 junction sequence reads were obtained and 90.8% of them were successfully mapped to the genome in K562 cells. In the GM12093 cells, 1,504,454 reads were obtained and 95.9% of them were successfully mapped. Relative association with the VEGFA promoter was analyzed with the use of the 4C-seqpipe tool [38]. For each cell type percent reads was calculated with 1-kb sliding widows at 50% overlap. The difference between the association of the ESSE site with the VEGFA promoter in GM12093 and K562 cells was evaluated by counting percent reads, out of all the reads, in 1Kb sliding windows (50% overlap) across a 100 kb region centered at the ESSE site. The P value was calculated according to the two-tailed Student’s paired t-test.

## Supporting Information

S1 Dataset Enriched groups of genes in the vicinity of cancer-hypermethylated ESSEs. (XLSX)

S1 Table Chromatin, DNA methylation and gene expression datasets used in the study. (PDF)

S2 Table Methylation fate in Jurkat T cell leukemia of sites highly methylated in normal T cells. (PDF)

S3 Table Enriched gene pathways adjacent to cancer-hypomethylated ESSEs. (PDF)

S1 Fig Developmental DNA methylation changes in regulatory sites. **A.** Numbers and percentages of methylation sites that lose, retain, or gain H3K4me1 marks during the development of the hematopoietic cell lineage. **B.** HCS versus ES methylation levels for enhancer sites that lose, retain or gain H3K4me1 marks in HSC versus ES. **C.** Same as B for promoter sites and H3K4me3 marks.
(PDF)

S2 Fig Developmental DNA methylation changes in promoter sites. **A.** Number and percentage of methylation sites that lose, maintain, or gain H3K4me3 marks between developmental stages. **B.** Methylation levels of late (Y-Axis) versus earlier (X-axis) developmental stages for promoter sites that lose (red), maintain (yellow), or gain (green) H3K4me3 signals during differentiation. Methylation levels in T and B-cells are given as well.
(PDF)

S3 Fig DNA methylation differences between ES, HSC and T cell samples. **A.** Methylation differences between a HSC sample and three ES samples for which methylation data were available, see S1 Table, in enhancer sites that lose, maintain, or gain H3K4me1 in HSC compared with ES. **B.** Same analysis as in A in T cell leukemia (Jurkat) compared with normal T cell samples.
(PDF)

S4 Fig Overlap between enhancer sites hypomethylated in cancer (Jurkat, at least 20% less methylated than CD4^+^ T cells), and enhancer sites that gain methylation in T cells compared with HSCs (CD4^+^ T cells at least 20% more methylated than HSC). (PDF)
