# Supplementary material for: Embryonic Stem Cell (ES)-Specific Enhancers Specify the Expression Potential of ES Genes in Cancer
Source: PLoS Genet. 2016 Feb 17;12(2):e1005840. doi: 10.1371/journal.pgen.1005840 (PMC4757527; doi:10.1371/journal.pgen.1005840)
Supplement: S1 Table — (PDF) [file pgen.1005840.s002.pdf]

S1 Table. Chromatin, DNA methylation and gene expression datasets used in the study.

	H3K4me1		H3K4me3		H3K27me3		DNA methylation		Gene expression	
**Sample**	#	Source	#	Source	#	Source	#	Source	#	Source
**ES**	6	Roadmap	9	Roadmap	6	Roadmap	3	GSE40909	3	GSE7332
**HSC ($CD34^+$)**	7	Roadmap	6	Roadmap	8	Roadmap	1	GSM1032771	2	GSE10325
**T-cell ($CD4^+$)**	6	Roadmap	7	Roadmap	6	Roadmap	6	GSE35069	9	GSE10325
**T-cell ($CD8^+$)**	5	Roadmap	5	Roadmap	5	Roadmap	6	GSE35069		
**B-cell ($CD19^+$)**	1	Roadmap	2	Roadmap	1	Roadmap	6	GSE35069	9	GSE10325
**Jurkat**							1	ENCODE	1	ENCODE
**K562**							1	ENCODE	1	ENCODE
**DLBCL**							28	TCGA	28	TCGA
**CRC**							290	TCGA	290	TCGA
**HCC**							207	TCGA	207	TCGA

Abbreviations: ES: Embryonic Stem Cells; **HSC:** Hematopoietic Stem Cells; **DLBCL:** Diffuse Large B-Cell Lymphoma; **CRC:** Colorectal Cancer; **HCC:** Hepatocellular Carcinoma; **Roadmap:** Roadmap Epigenomics Project; **GSE#:** Accession number in the Gene Expression Omnibus (GEO); **ENCODE:** Encyclopedia of DNA Elements.
