# Supplementary material for: Embryonic Stem Cell (ES)-Specific Enhancers Specify the Expression Potential of ES Genes in Cancer
Source: PLoS Genet. 2016 Feb 17;12(2):e1005840. doi: 10.1371/journal.pgen.1005840 (PMC4757527; doi:10.1371/journal.pgen.1005840)
Supplement: S2 Table — (PDF) [file pgen.1005840.s003.pdf]

S2 Table. Methylation fate in Jurkat T-cell leukemia of sites that are highly methylated in normal T-cells.

Non-promoter sites that are methylated (>70%) in T-cells		Hypomethylation in T-cell leukemia			
**Class**	**#**	**Fraction of class** (%)	**Fold-ratio** (*versus* all)	**P-value** (*versus* all)	**Average cancer-normal difference**
**All**	130,794	18.2			-10.2%
**ESSEs**	35,392	22.6	1.24	<1e-20	-12.3%
**ESSEs unmethylated in ES**	3,451	26.5	1.46	<1e-20	-12.5%
**Non-ESSE sites unmethylated in ES**	2,825	19.0	1.04	0.14	-8.1%
**Constitutive enhancers unmethylated in ES**	1,266	13.1	0.72	1.9e-3	-4.1%
