# Supplementary material for: Embryonic Stem Cell (ES)-Specific Enhancers Specify the Expression Potential of ES Genes in Cancer
Source: PLoS Genet. 2016 Feb 17;12(2):e1005840. doi: 10.1371/journal.pgen.1005840 (PMC4757527; doi:10.1371/journal.pgen.1005840)
Supplement: S3 Table — (PDF) [file pgen.1005840.s004.pdf]

S3 Table. Enriched groups of genes adjacent to cancer-hypomethylated ESSEs.

Common pathway	Rank	P-val.	FDR Q-val.	Fold Enrich.	Foreground hits	Background hits	FgSetTermCov	Foreground genes	Total Genes
Syndecan-4-mediated signaling events	1	3.E-08	4.E-05	1.61	129	714	0.03	64	209
VEGF and VEGFR signaling network	2	7.E-08	6.E-05	1.26	456	3239	0.11	258	1304
Signaling events mediated by VEGFR1 and VEGFR2	3	9.E-08	5.E-05	1.26	447	3172	0.11	255	1296
Wnt signaling network	4	3.E-07	1.E-04	1.58	122	692	0.03	58	200
Proteoglycan syndecan-mediated signaling events	5	3.E-07	9.E-05	1.24	458	3293	0.11	265	1345
Wnt	6	5.E-07	1.E-04	1.79	74	369	0.02	30	82
Noncanonical Wnt signaling pathway	7	7.E-07	2.E-04	1.59	108	606	0.03	51	182
IL5-mediated signaling events	8	8.E-07	2.E-04	1.24	436	3146	0.11	251	1292
PAR1-mediated thrombin signaling events	9	9.E-07	2.E-04	1.24	435	3141	0.10	252	1299
Thrombin/protease-activated receptor (PAR) pathway	10	9.E-07	1.E-04	1.24	435	3142	0.10	252	1300
Glypican pathway	11	1.E-06	1.E-04	1.23	469	3420	0.11	269	1338
Sphingosine 1-phosphate (S1P) pathway	12	1.E-06	1.E-04	1.23	442	3201	0.11	255	1310
Alpha9 beta1 integrin signaling events	13	1.E-06	1.E-04	1.23	437	3164	0.11	254	1307
Signaling events mediated by Hepatocyte Growth Factor Receptor (c-Met)	14	1.E-06	2.E-04	1.23	433	3138	0.10	251	1293
Glypican 1 network	15	2.E-06	2.E-04	1.23	439	3189	0.11	255	1299
Syndecan-1-mediated signaling events	16	2.E-06	2.E-04	1.23	434	3149	0.10	252	1300
Arf6 downstream pathway	17	2.E-06	1.E-04	1.23	431	3125	0.10	250	1288
S1P1 pathway	17	2.E-06	1.E-04	1.23	431	3125	0.10	250	1288
mTOR signaling pathway	17	2.E-06	1.E-04	1.23	431	3125	0.10	250	1288
Class I PI3K signaling events mediated by Akt	17	2.E-06	1.E-04	1.23	431	3125	0.10	250	1288
Arf6 trafficking events	17	2.E-06	1.E-04	1.23	431	3125	0.10	250	1288
ErbB1 downstream signaling	17	2.E-06	1.E-04	1.23	431	3125	0.10	250	1288
Signaling events mediated by focal adhesion kinase	17	2.E-06	1.E-04	1.23	431	3125	0.10	250	1288
Arf6 signaling events	17	2.E-06	1.E-04	1.23	431	3125	0.10	250	1288
Insulin Pathway	17	2.E-06	1.E-04	1.23	431	3125	0.10	250	1288
Urokinase-type plasminogen activator (uPA) and uPAR-mediated signaling	17	2.E-06	1.E-04	1.23	431	3125	0.10	250	1288
Internalization of ErbB1	17	2.E-06	1.E-04	1.23	431	3125	0.10	250	1288
Class I PI3K signaling events	17	2.E-06	1.E-04	1.23	431	3125	0.10	250	1288
EGF receptor (ErbB1) signaling pathway	17	2.E-06	1.E-04	1.23	431	3125	0.10	250	1288
PDGFR-beta signaling pathway	17	2.E-06	1.E-04	1.23	431	3125	0.10	250	1288
LKB1 signaling events	31	2.E-06	9.E-05	1.23	435	3159	0.10	251	1308
IFN-gamma pathway	32	2.E-06	8.E-05	1.23	432	3135	0.10	251	1297
EGFR-dependent Endothelin signaling events	33	2.E-06	8.E-05	1.23	431	3128	0.10	250	1289
GMCSF-mediated signaling events	33	2.E-06	8.E-05	1.23	431	3128	0.10	250	1293
PDGF receptor signaling network	35	2.E-06	8.E-05	1.23	435	3162	0.10	253	1294
IL3-mediated signaling events	36	2.E-06	8.E-05	1.23	431	3130	0.10	250	1296
ErbB receptor signaling network	37	2.E-06	1.E-04	1.22	439	3203	0.11	257	1311
IGF1 pathway	38	2.E-06	1.E-04	1.23	431	3138	0.10	250	1291
Nectin adhesion pathway	39	3.E-06	1.E-04	1.23	432	3148	0.10	251	1295
Plasma membrane estrogen receptor signaling	40	3.E-06	1.E-04	1.23	431	3143	0.10	250	1300
TRAIL signaling pathway	41	3.E-06	1.E-04	1.22	439	3209	0.11	255	1327
Integrin-linked kinase signaling	42	3.E-06	1.E-04	1.32	242	1634	0.06	126	656
Endothelins	43	3.E-06	1.E-04	1.22	438	3203	0.11	254	1306
Regulation of CDC42 activity	44	1.E-05	5.E-04	1.27	283	1995	0.07	152	772
CDC42 signaling events	45	1.E-05	5.E-04	1.27	281	1980	0.07	151	759
Beta1 integrin cell surface interactions	46	2.E-05	6.E-04	1.19	461	3447	0.11	269	1352
Integrin family cell surface interactions	47	2.E-05	7.E-04	1.19	467	3503	0.11	273	1379
AP-1 transcription factor network	48	2.E-05	7.E-04	1.30	225	1546	0.05	119	623
Validated targets of C-MYC transcriptional activation	49	5.E-05	2.E-03	1.97	36	163	0.01	17	78
Glypican 3 network	50	1.E-04	5.E-03	1.38	123	797	0.03	59	206
Regulation of nuclear beta catenin signaling and target gene transcription	51	2.E-04	5.E-03	1.51	77	455	0.02	37	135
N-cadherin signaling events	52	2.E-04	7.E-03	1.37	122	797	0.03	63	251
Canonical Wnt signaling pathway	53	3.E-04	8.E-03	1.48	79	478	0.02	39	155
Calcineurin-regulated NFAT-dependent transcription in lymphocytes	54	3.E-04	9.E-03	2.23	21	84	0.01	11	50
