# Supplementary material for: Embryonic Stem Cell (ES)-Specific Enhancers Specify the Expression Potential of ES Genes in Cancer
Source: PLoS Genet. 2016 Feb 17;12(2):e1005840. doi: 10.1371/journal.pgen.1005840 (PMC4757527; doi:10.1371/journal.pgen.1005840)

A

Non-promoter methylation sites (n=179,910)

**From**	**To**	**H3K4me1** Lose	Retain	Gain	**Fraction of H3K4me1 sites (%)** Lose	Retain	Gain
**ES**	**HSC**	49031	20380	10314	61.5	25.6	12.9
**ES**	**T ($CD4^+$)**	49455	19956	11375	61.2	24.7	14.1
**HSC**	**T ($CD4^+$)**	11802	18892	12439	27.4	43.8	28.8
**HSC**	**T ($CD8^+$)**	11422	19272	13735	25.7	43.4	30.9
**HSC**	**B ($CD19^+$)**	14155	16539	6780	37.8	44.1	18.1
**T ($CD4^+$)**	**B ($CD19^+$)**	15754	15577	7742	40.3	39.9	19.8
**T ($CD4^+$)**	**T ($CD8^+$)**	3331	28000	5007	9.2	77.1	13.8

B

Enhancers

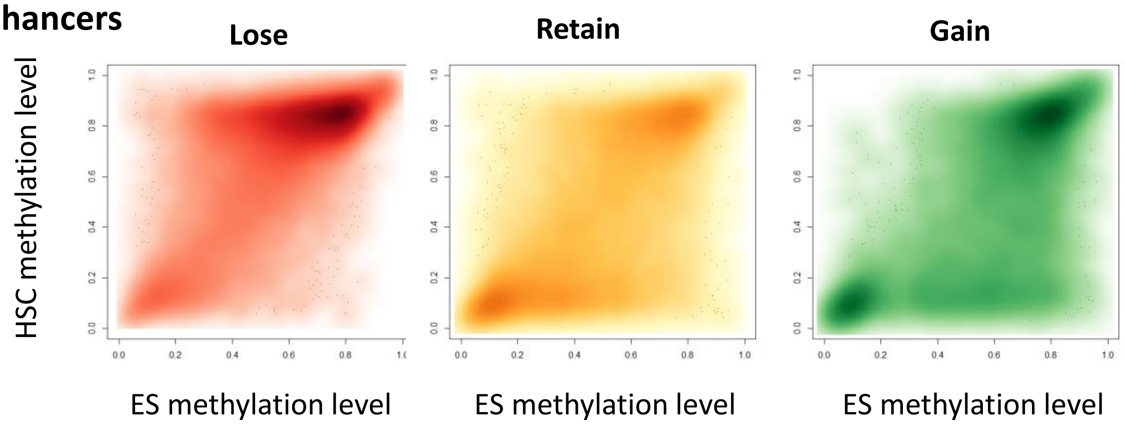

C

Promoters

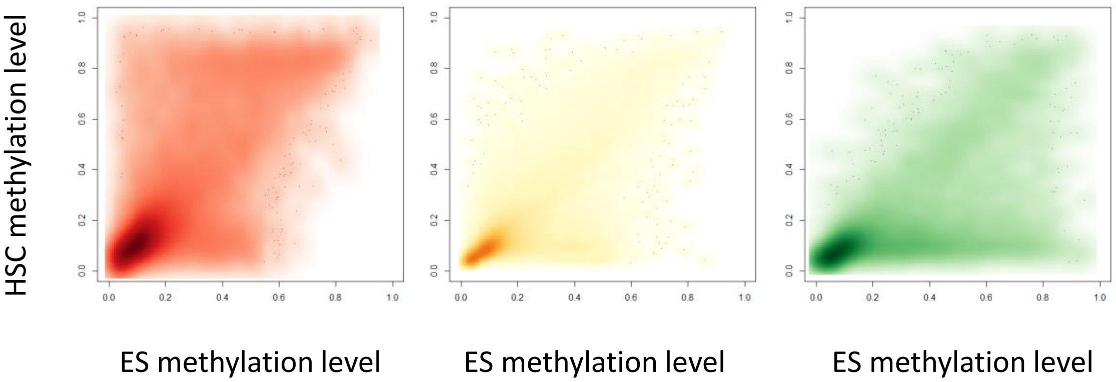

Supplement: S1 Fig — A. Numbers and percentages of methylation sites that lose, retain, or gain H3K4me1 marks during the development of the hematopoietic cell lineage. B. HCS versus ES methylation levels for enhancer sites that lose, retain or gain H3K4me1 marks in HSC versus ES. C. Same as B for promoter sites and H3K4me3 marks. (PDF) [file pgen.1005840.s005.pdf]
