# Supplementary material for: Embryonic Stem Cell (ES)-Specific Enhancers Specify the Expression Potential of ES Genes in Cancer
Source: PLoS Genet. 2016 Feb 17;12(2):e1005840. doi: 10.1371/journal.pgen.1005840 (PMC4757527; doi:10.1371/journal.pgen.1005840)

A

Promoter H3K4me3 sites (n=229,306)

From	To	H3K4me3 Red	H3K4me3 Yellow	H3K4me3 Green	% of H3K4me3 sites Red	% of H3K4me3 sites Yellow	% of H3K4me3 sites Green
**ES**	**HSC**	19269	106049	14859	13.7	75.7	10.6
**ES**	**T (CD4+)**	28831	96487	15157	20.5	68.7	10.8
**HSC**	**T (CD4+)**	18927	101981	9663	14.5	78.1	7.4
**HSC**	**B (CD19+)**	14665	106243	10650	11.1	80.8	8.1
**T (CD4+)**	**B (CD19+)**	14510	102383	9261	11.5	81.2	7.3
**T (CD4+)**	**T (CD8+)**	5685	105959	2945	5.0	92.5	2.6

B

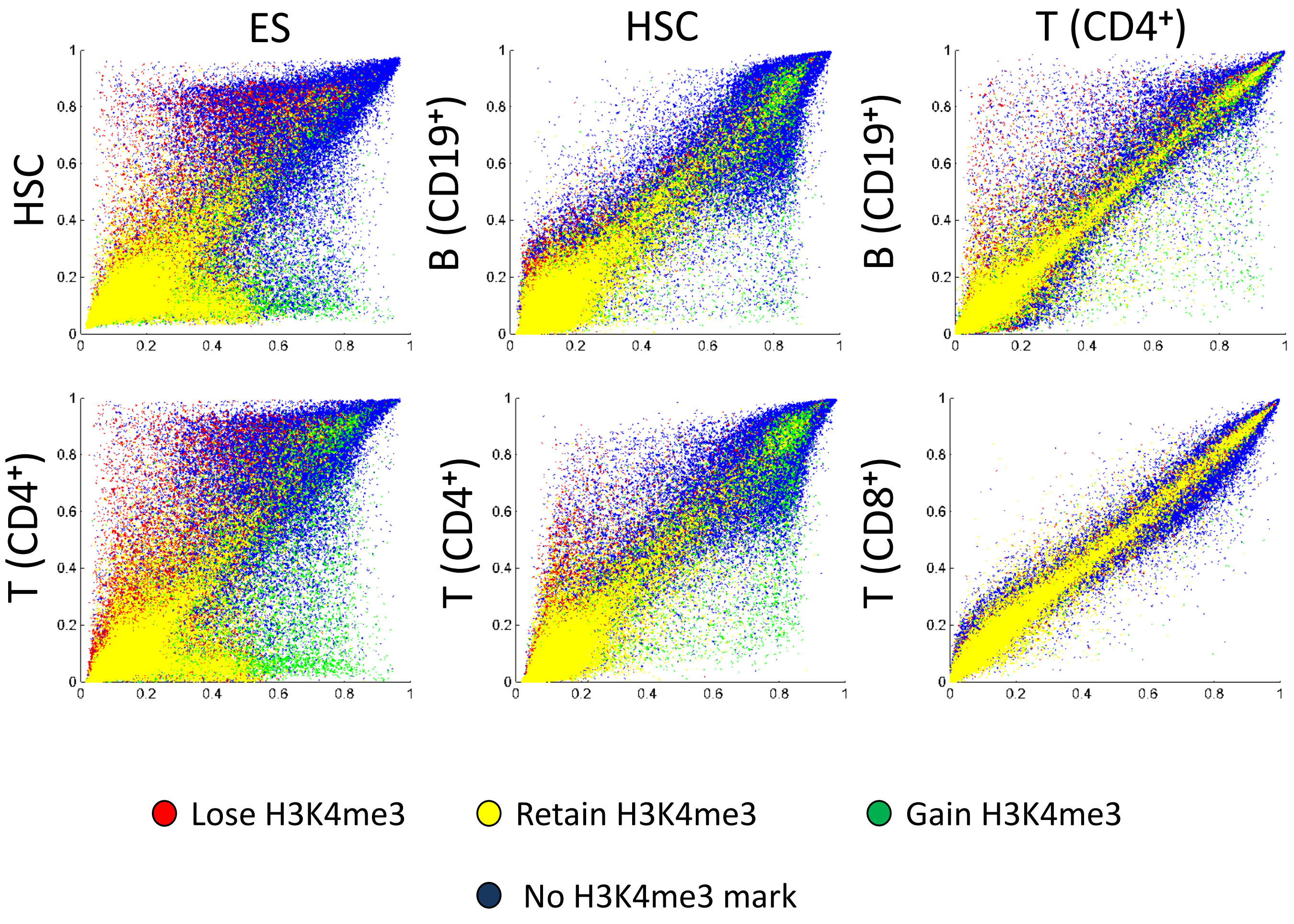

Supplement: S2 Fig — A. Number and percentage of methylation sites that lose, maintain, or gain H3K4me3 marks between developmental stages. B. Methylation levels of late (Y-Axis) versus earlier (X-axis) developmental stages for promoter sites that lose (red), maintain (yellow), or gain (green) H3K4me3 signals during differentiation. Methylation levels in T and B-cells are given as well. (PDF) [file pgen.1005840.s006.pdf]
