# Supplementary figures and images for: Embryonic Stem Cell (ES)-Specific Enhancers Specify the Expression Potential of ES Genes in Cancer
Source: PLoS Genet. 2016 Feb 17;12(2):e1005840. doi: 10.1371/journal.pgen.1005840 (PMC4757527; doi:10.1371/journal.pgen.1005840)

## HSC vs. ES

**A**

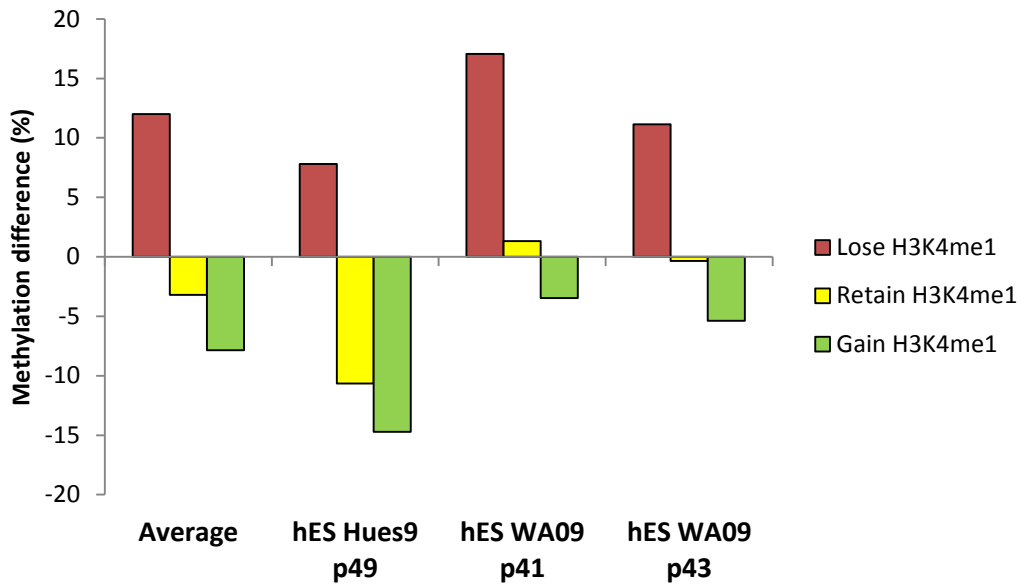

## T cell leukemia vs. T cells

**B**

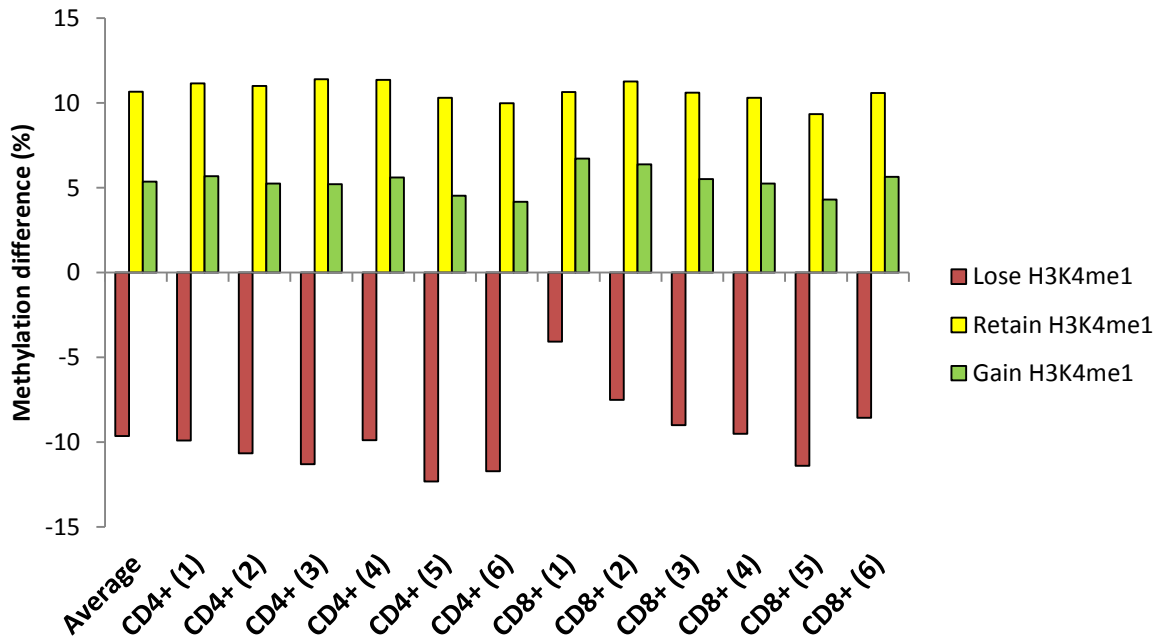

Supplement: S3 Fig — A. Methylation differences between a HSC sample and three ES samples for which methylation data were available, see S1 Table, in enhancer sites that lose, maintain, or gain H3K4me1 in HSC compared with ES. B. Same analysis as in A in T cell leukemia (Jurkat) compared with normal T cell samples. (PDF) [file pgen.1005840.s007.pdf]
