# Supplementary material for: Embryonic Stem Cell (ES)-Specific Enhancers Specify the Expression Potential of ES Genes in Cancer
Source: PLoS Genet. 2016 Feb 17;12(2):e1005840. doi: 10.1371/journal.pgen.1005840 (PMC4757527; doi:10.1371/journal.pgen.1005840)
Supplement: S4 Fig — (PDF) [file pgen.1005840.s008.pdf]

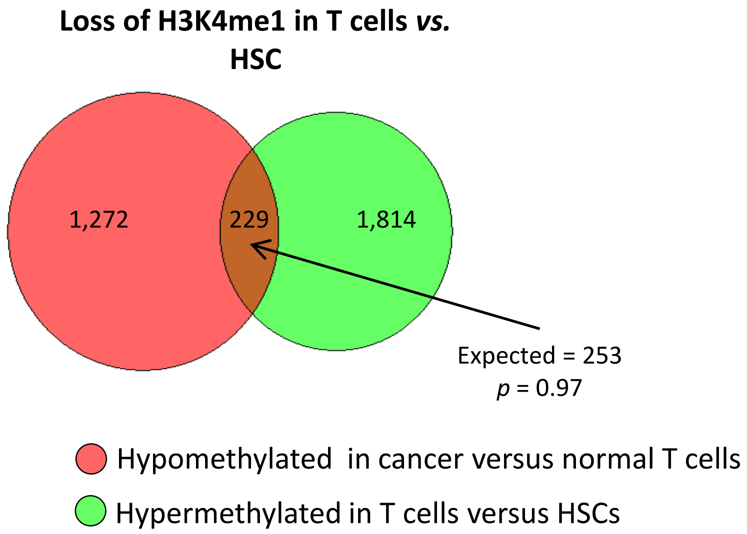
Loss of H3K4me1 in T cells *vs.* HSC
1,272
229
1,814
Expected = 253
p = 0.97
Hypomethylated in cancer versus normal T cells
Hypermethylated in T cells versus HSCs
